# Multiple mutations of lung squamous cell carcinoma shared common mechanisms

**DOI:** 10.18632/oncotarget.13190

**Published:** 2016-11-07

**Authors:** Qianping Li, Junyi Hou, Zhaoyan Hu, Biao Gu, Yan Shi

**Affiliations:** ^1^ Department of Cardiothoracic Surgery, Shanghai University of Medicine & Health Sciences Shanghai Sixth People's Hospital East Campus, Shanghai, PR China; ^2^ Department of Gastroenterology, Shanghai University of Medicine & Health Sciences Shanghai Sixth People's Hospital East Campus, Shanghai, PR China; ^3^ Hangzhou Cancer Institute, Hangzhou cancer hospital, Hangzhou City, PR China; ^4^ Department of Thoracic Surgery, Huai'an First People's Hospital, Nanjing Medical University, Huai'an, Jiangsu, China; ^5^ Department of Emergency, The Affiliated Huai'an Hospital of Xuzhou Medical University and The Second People's Hospital of Huai'an, Huai'an, China

**Keywords:** lung squamous cell carcinoma, mutation, the cancer genome atlas, mRNA

## Abstract

Lung squamous cell carcinoma (LUSC) is a subtype of non-small cell lung cancers which is the cause of 80% of all lung cancer deaths. The genes that highly mutated in patients with LUSC and their roles played in the tumorigenesis remains unknown. Data of patients with Lung squamous cell carcinoma (LUSC) were retrieved from The Cancer Genome Atlas (TCGA). Differentially expressed genes were identified between control and cancer samples. Patients and controls can be separated by mRNA expression level showing that the between-group variance and totally 1265 genes were differentially expressed between controls and patients. Top genes whose mutations highly occurred in patients with LUSC were identified, most of these genes were shown to be related with tumorigenesis in previous studies. All of the genes mostly mutated were independently correlated with expression levels of all genes. These mutations did not show the trend of co-occurrence. However, the influenced gene of these mutations had overlaps. After studying the intersection of these genes, a group of shared genes were identified. The shared pathways enriched which played critical role in LUSC were identified based on these shared genes. Different mutations had contribution to the progression of LUSC. Though these genes involved different specific mechanisms, most of them may share a common mechanism which is critical for LUSC. The results may suggest a neglected mechanism and also indicate a potential target for therapies.

## INTRODUCTION

Lung cancer is the most common cause of deaths that related to cancer in the world. On the meantime, non–small cell lung cancers, causing about 80% of all lung cancer deaths in the United States, is the most frequent form of lung cancer [1]. Lung squamous cell carcinoma (LUSC) is one of the primary subtypes of non-small cell lung cancers.

Even when potentially curative surgery were carried out, about 40% of patients with LUSC will relapse within 5 years [2]. While most cancers have a steady increase in survival in the past decades, lung cancers have slow advance in this aspect, whose currently 5-year relative survival is only 18% and 7%, respectively [3]. These low rates are, to some extent, associated with the fact that more than 50% of patients with LUSC are diagnosed at a distant stage [3].

A lot of studies have been carried out to improve patients' prognosis. Wilkerson, Yin [4] categorized LUSC into four subtypes using mRNA expression, which could be used to hint survival outcomes. Cancer Genome Atlas Research [5] identified potential therapeutic targets like pathways included NFE2L2 and KEAP1, squamous differentiation genes, phosphatidylinositol-3-OH kinase pathway genes and so forth. However, considering the high prevalence and the poor prognosis of LUSC, it is worthwhile to study more about it. This paper aims to figure out the important pathways and mechanisms that lead to the LUSC.

## RESULTS

### Differentially expressed genes between controls and patients

Differentially expressed genes between controls and patients were identified using R package EBSeq [6]. Totally 1265 genes were differentially expressed.

**Figure 1:**
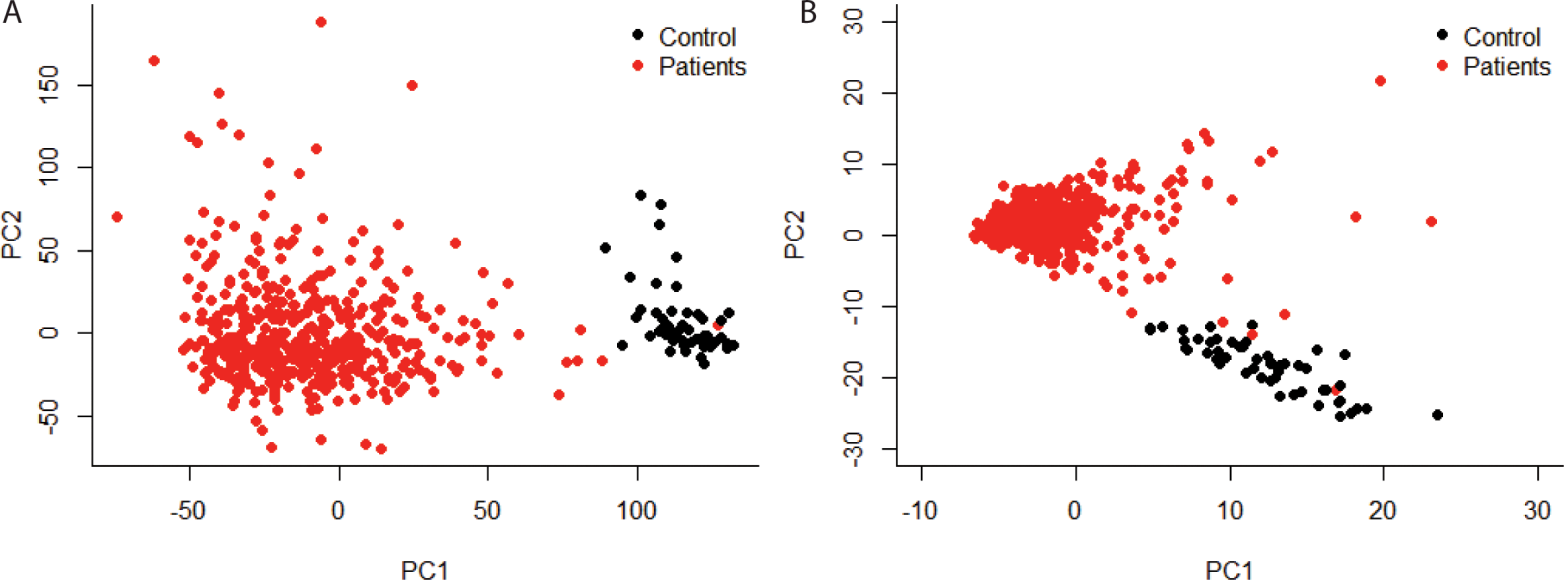
Principal component analysis of gene expression level Only principal component 1 and 2 were shown. (**A**) all the genes were used; (**B**) only genes that were differentially expressed between patients and control were used.

Principal component analysis (PCA) was carried out on the mRNA expression level among all genes and only the differentially genes. Even with the whole gene group, mRNA expression level can separate patients with control group (Figure 1A), which demonstrated that the biggest variance among all the samples was whether the sample had LUSC. In this case, principal component 1 and 2 accounted for 9.6% and 4.8% of the variance, respectively. With only the differentially expressed genes (Figure 1B), though the variance represented by PC1 and PC2 decreased, the within-group variance also decreased.

The GO terms and pathways related with these genes were summarized in Table 1. These genes shared only a few pathway or COG terms. This suggested that a lot of mechanisms resulted in the difference from patients to control. These mechanisms masked other pathway or terms. When considering the enriched pathway, the olfactory transduction was significantly enriched which can be explained by the involvement of lung in the olfactory transduction. Also, neuroactive ligand-receptor interaction pathway was usually related with cancer progression [7]. Interestingly, systemic lupus erythematosus was enriched, which seemed unrelated to LUSC. However, in most patients with systemic lupus erythematosus, lung involvement is a known complication. Also lupus patients experienced an elevated risk of different cancers including lung cancer.

**Table 1:** The pathway and COG enrichment of genes differentially expressed genes between patients and controls

Category	Term	*P*-Value	Benjamini
Pathway	Neuroactive ligand-receptor interaction	1.40E-14	1.70E-12
	Olfactory transduction	1.80E-10	1.10E-08
	Systemic lupus erythematosus	4.60E-04	1.90E-02
	Secondary metabolites biosynthesis, transport, and catabolism	1.50E-03	1.90E-02
COG	Signal transduction mechanisms / Cytoskeleton / Cell division and chromosome partitioning / General function prediction only	2.70E-02	1.70E-01
	Amino acid transport and metabolism	4.20E-02	1.70E-01
	Lipid metabolism	9.30E-02	2.70E-01

### Mutations occurred in patients with LUSC

Top genes whose mutations highly occurred in patients with LUSC were identified with the somatic mutations datasets from TCGA. The overall results were shown in Table 2. Most of these genes were shown to be related with tumorigenesis in previous studies mathematically or biologically. Here, abParts stood for parts of antibodies which are mostly variable regions. Due to the fact it was not a gene, it was excluded in the following analysis. On the meantime, it is quite common for a variable regions to mutate. *PCDHGC5, PCDHAC2, SPTA1, XIRP2 and FLG* seemed unrelated with cancer based the reference study.

**Table 2:** The summary of top genes that are frequently mutated in patients with LUSC

Symbol	Non-synonymous Mutations	Patients	Experimental Evidences
TTN	337	129	Mathematically based prediction without biological evidence [9, 10]^%^
abParts^$^	221	110	
TP53	147	141	Tumor protein
MUC16	142	77	Tumor antigen
CSMD3	124	81	Contributing to lung tumorigenesis [13]
RYR2	120	76	Related with apoptosis and carcinogenesis [14, 15]
LRP1B	103	69	Related with lung cancer^%^
PCDHGC5	103	69	
PCDHAC2	100	53	
USH2A	93	67	Mutations are frequent in cancer [9]^%^
ZFHX4	90	65	Related with glioblastoma [16]
SYNE1	70	52	Methylated in lung cancer [17]^%^
RYR3	54	41	Regulator of breast cancer cell [18]
MLL2	52	43	Associated with gain-of-function p53 mutations [19]
SPTA1	51	40	
FAM135B	49	37	Promote malignancy of oesophageal squamous cell carcinoma [20]
XIRP2	48	33	
COL11A1	46	35	Promote tumor progression in ovarian cancer [21]
SI	46	36	Related with cancer development^%^
FLG	45	36	

$ abParsts meant parts of antibodies which are mostly variable regions.

% Reports showed that mutations and/or mRNA expression of these genes were related to tumors but no experiments showed that knockout or mutations would lead to cancer progression.

The other genes were all shown to have connection with cancer progression. However, some of them were only related to cancer without evidences whether they would lead to cancer progression, or they were mutated led by cancer or other genes. These genes were marked as red in the Table 2. Our attention was mainly put on the genes that had proved to have contribution to tumorigenesis. These genes are possibly important driver genes leading to LUSC, instead of just passenger genes.

Another interesting point is that average of mutations of a certain gene in was different. For example, TTN was mutated in 129 patients and every patient had 2.61 TTN mutations in average. On the other hand, every patient had 1.04 mutations of TP53 in average. The ratio of non-synonymous mutations and patients might suggest that whether one mutation in this gene is critical to tumorigenesis. This result also showed that any mutation on TP53, together mutations on other genes, might lead to cancer. In this study, MLL2 also had a relative low ratio of non-synonymous mutations and patients.

### Mutation patterns and global gene expression

**Figure 2:**
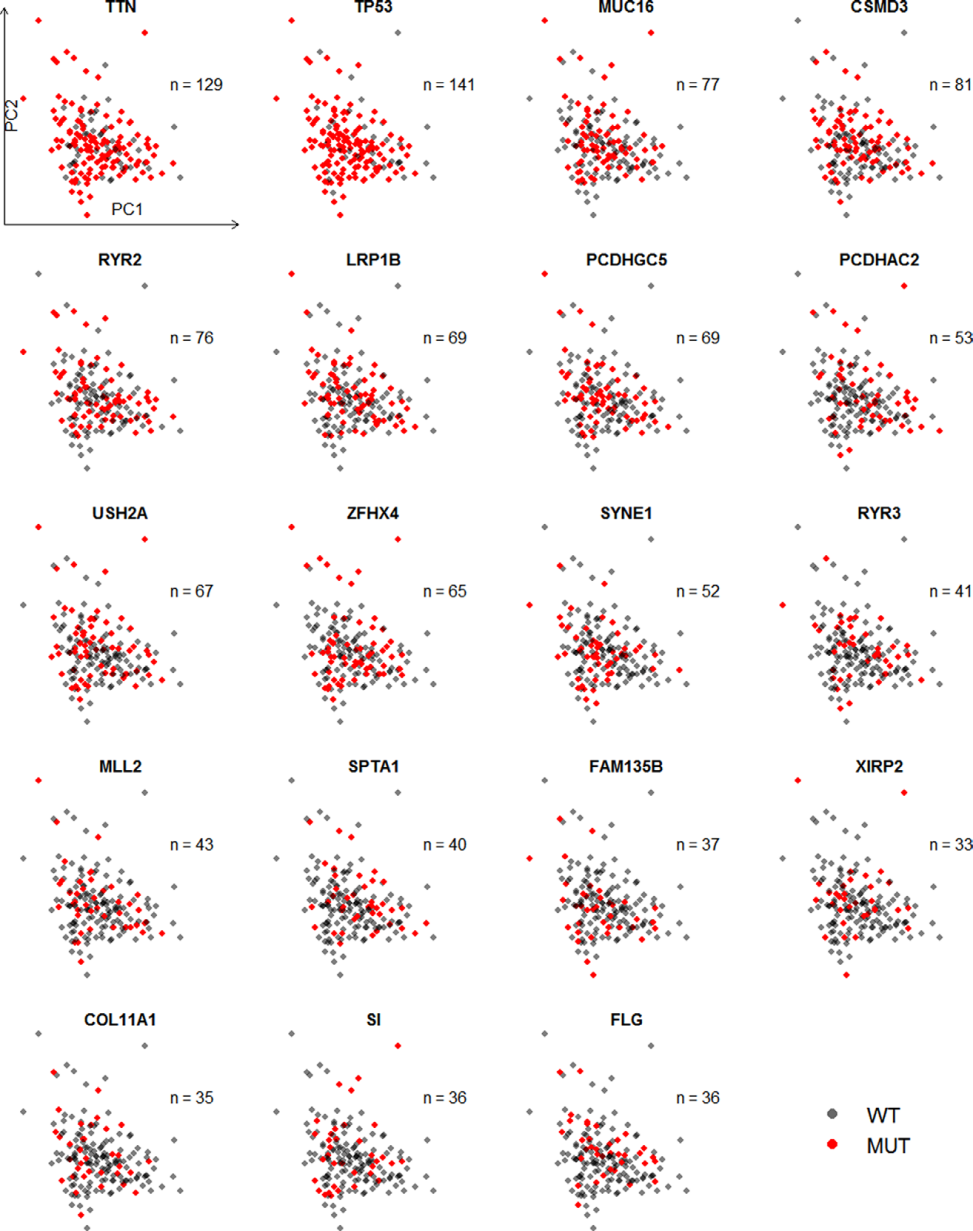
Scatter plot of the first two principal components of expression levels of all available genes with mutation status of 19 genes This principal component analysis involved all 501 patients with mRNA expression level and 51 controls, though controls and patients without mutations' information were excluded in the plot.

Strikingly, all of the genes mostly mutated were independently correlated with expression levels of all genes (Figure 2) and 1261 differentially expressed genes (data not shown). However, Gerstung, Pellagatti [8] found that the principal component values of target genes varied widely across the different mutations or indels in patients with myelodysplastic syndromes. It suggested the different mutation patterns between LUSC and chronical blood cancer.

**Figure 3:**
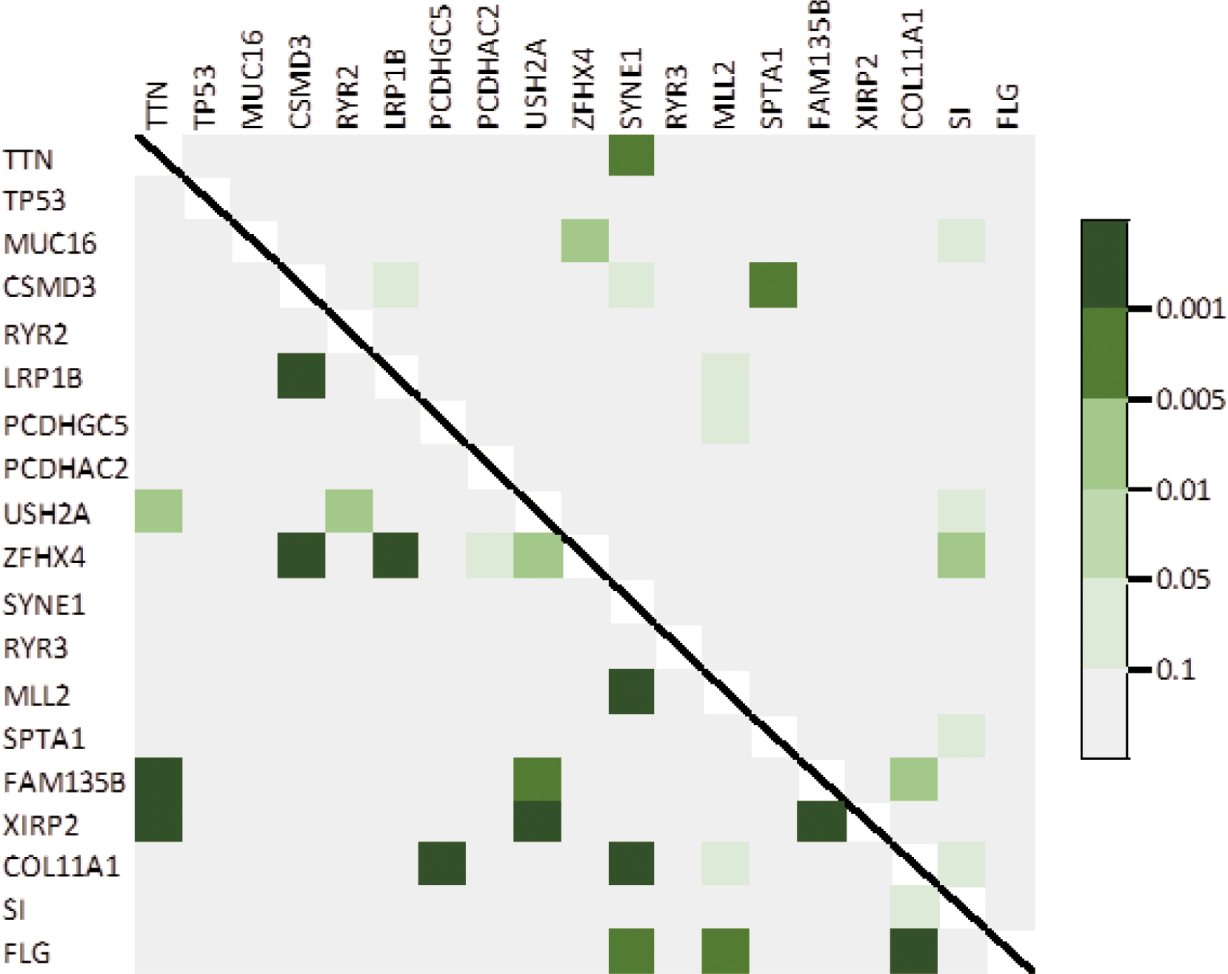
Heatmap of observed pairwise mutation patterns (upper triangle) and the pairwise correlation of expression level (lower triangle)

Figure 3 showed the pairwise heatmap between top genes. The upper triangle showed that whether mutations of two different genes co-occurred and the lower triangle showed that whether the expression levels of two different genes were correlated. TTN and SYNE1, CSMD3 and SPTA1, MUC16 and ZFHX4, ZFHX4 and SI, and FAM135B and COL11A1 had the highest possibilities that their mutations occur simultaneously. But mainly, these mutations did not occur on the same time. Combining the expression levels of these genes, it suggested that there was no relation between mutation and expression. The mutation on one gene may not influence the expression of it.

### Effects of mutations on expression

**Figure 4:**
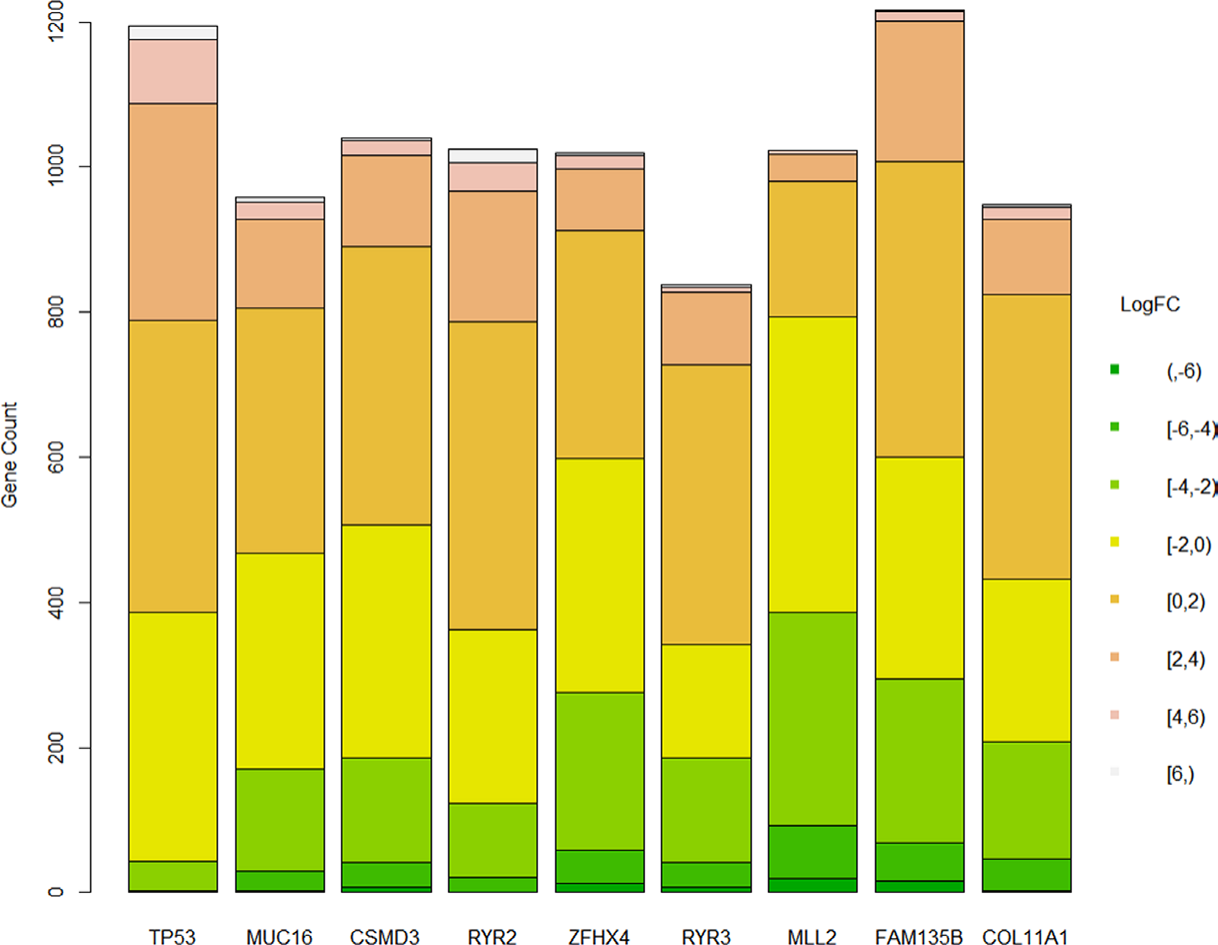
Difference of logarithm fold changes on genes that were differentially expressed whether a gene was mutated

After figuring out all genes that were differentially expressed between groups where a certain gene was mutated or not, the summary of the logarithm fold changes was plotted as a barplot (Figure 4). There seemed no significant difference between different genes.

After studying the pathway involved, it should that different genes had some common pathway, including neuroactive ligand-receptor interaction, retinol metabolism, drug metabolism, steroid hormone biosynthesis and so forth. The fully list is not shown here. It is quite interesting because there seemed no common patterns among these genes. They were independently correlated with expression levels of all genes and their mutation did not show co-occurrence.

### Functional analysis of mutation-related expression change

After studying the intersection of these differentially genes of different genes, a group of shared gene were identified. Only the genes that were shared by at least four genes were extracted here. Pathway enrichment was carried out on these group of genes (Table 3). The shared pathway enrichment results of each genes were close to this table as expected. This table showed a shared mechanisms and pathways in patients with different gene mutations and suggested the important roles that these pathways played in LUSC.

**Table 3:** Pathway enrichment for shared differentially expressed genes

Term	*P*-Value	Benjamini
Neuroactive ligand-receptor interaction	3.20E-14	4.10E-12
Drug metabolism	6.80E-09	4.30E-07
Retinol metabolism	1.40E-07	4.40E-06
Metabolism of xenobiotics by cytochrome P450	5.20E-07	1.30E-05
Steroid hormone biosynthesis	1.10E-05	2.30E-04
Androgen and estrogen metabolism	8.90E-05	1.60E-03
Tyrosine metabolism	3.20E-04	5.00E-03
Ascorbate and aldarate metabolism	4.30E-04	6.00E-03
Pentose and glucuronate interconversions	5.80E-04	7.30E-03
Starch and sucrose metabolism	1.30E-03	1.50E-02
Maturity onset diabetes of the young	2.80E-03	2.90E-02

## DISCUSSION

This study identified genes which were highly mutated in patients with LUSC. Among all of the genes that had the most mutations, some of them lack evidence to link them with the cancer. One reason may be that there are high possibility that mutations occur on these genes, even though they themselves did not have impact on the progression of LUSC. For example, abParts was identified, which is the most variable regions in antibodies. It, certainly, had higher mutation rate. On the other hand, the length of the gene has an impact. The longer the gene is, the higher possibility that a mutation occurs is. Proteins of TTN and MUC16 are extremely long, respectively 34350 and 22152. This may induce bias in the identification of mutations. However, basically, the identified genes that are highly mutated were reported to be related to cancer or lung cancer in the previous studies and also some of them may play roles as driver genes that result in LUSC. Here TTN and SYNE1 were used as an example. Kim, Hong [9] found that TTN had dominant frequencies in eleven different tumors and Greenman, Stephens [10] stated that its functions were compatible with a role in oncogenesis. SYNE1 expressed in skeletal and smooth muscle and localizes to the nuclear membrane, but a lot of studies reported that missense mutations, silent mutations, nonsense mutations, and frameshift deletions on SYNE1 were observed in colon cancer, stomach cancer, breast cancer and so forth [11, 12].

These genes showed independently pattern with the expression level of all genes. Also, there seemed no co-occurrence between these genes. However, the differentially expressed genes between patients with or without a certain gene have an intersection. 741 out of 3022 genes, which were differentially expressed in any gene, appeared in at least four groups. It suggested that the mechanisms that the mutation of these genes led to LUSC may have an overlap. The pathways enriched using the shared genes may be extremely important.

Among these pathways, only neuroactive ligand-receptor interaction was enriched using differentially expressed genes between patients and controls. Interestingly, most of these pathways are related to metabolism, including metabolism of retinol, xonobiotics, androgen and estrogen, tyrosine, ascorbate and aldarate, and starch and sucrose. It suggests that any mutation on the driver genes may lead to the different patterns of metabolism, while LUSC itself has a smaller impact on the metabolism.

## MATERIALS AND METHODS

### TCGA lung squamous cell carcinoma dataset

Clinical information, level-3 data of microarray and mutation information from patients with lung squamous cell carcinoma were retrieved from The Cancer Genome Atlas (TCGA). This data set contains 504 patients, within which 501 and 497 patients, respectively, had mRNA and mutations information. On the meantime, mRNA expression levels of another 51 samples from normal solid tissue were used as control.

### Differentially expressed genes

Differentially expressed genes were found using R packages EBSeq [6] between control and cancer samples. Biological functions were summarized using Clusters of Orthologous Groups (COG) terms [22] and pathway enrichment using DAVID Bioinformatics Resources 6.7 [23]. Principal component analysis (PCA) was carried out on the mRNA expression level among all genes, as well as among only the differentially genes.

### Mutations identification

Top 20 genes with most mutations in patients were identified using mutation information. References searching was carried out to figure out whether these genes were related to lung squamous cell carcinoma in the previous reports. The experimentally proved genes where had impact on the cancer progression were focused on the following analysis.

### Mutation patterns with mRNA expression

Global analysis Scatter plot of the first two principal components was plotted [8] was carried out to find out whether mutations of specific genes occurred among some group of patients, whether certain mutations occurred simultaneously in patients, and whether the expression levels of these most mutated genes were correlated with other.

### Effects of mutations on expression

Patients were separated into two groups that whether the patient had mutations of a certain gene [8] using EBseq package. In this study, except for abParts, which stood for the parts of antibodies which are mostly variable regions, the differentially expressed genes between two groups separated by another 19 genes were found. COG terms and pathway enrichment were carried out based on the genes identified by each mutation.

### Shared differentially expressed genes

Genes that differentially expressed in groups whether a certain gene was mutated were shared by different groups. Using genes that shared by at least four group were extracted and GO term and pathway enrichment were carried out as well.
